# Transition of a vestibular schwannoma to a malignant peripheral nerve sheath tumor with loss of H3K27 trimethylation after radiosurgery—a case report and review of the literature

**DOI:** 10.1007/s10143-021-01620-3

**Published:** 2021-08-15

**Authors:** Felix Behling, Imane Bersali, Antonio Santacroce, Johann Hempel, Kosmas Kandilaris, Jens Schittenhelm, Marcos Tatagiba

**Affiliations:** 1 Department of Neurosurgery, Eberhard-Karls University of Tübingen, Hoppe-Seyler Street 3, 72076 Tübingen, Germany; 2 Comprehensive Cancer Center Tübingen, Eberhard-Karls University, Tübingen, Germany; 3 Department of Neurosurgery, St. Barbara-Klinik Hamm-Heessen, Hamm, Germany; 4 Department of Diagnostic and Interventional Neuroradiology, Eberhard-Karls University, Tübingen, Germany; 5 Department of Neuropathology, Eberhard-Karls University, Tübingen, Germany

## Introduction

Vestibular schwannomas (VS) are benign slow-growing tumors arising from the vestibular nerve and are the most common intracranial nerve sheath tumors [1]. Nonetheless, due to the location in the internal auditory canal and the cerebellopontine angle, several neurovascular structures are at risk with tumor progression. Through microsurgical resection under intraoperative neuromonitoring in specialized skull base centers, most VS can be safely resected [2, 3]. As an alternative treatment, radiation therapy and radiosurgery have produced good tumor control rates for smaller vestibular schwannomas [4]. However, like microsurgery, radiation is not without risk. Besides the risk of radiotoxicity to cranial nerves and the brainstem [5], malignant transformation after radiotherapy has been described in several cases [6–8]. We now present a case of a vestibular schwannoma that received multimodal treatments including two sessions of gamma knife radiosurgery and transformed into a malignant peripheral nerve sheath tumor (MPNST). The clinical course as well as the histopathological evaluation is described together with a review of the current literature.

## Clinical summary

**Fig. 1:**
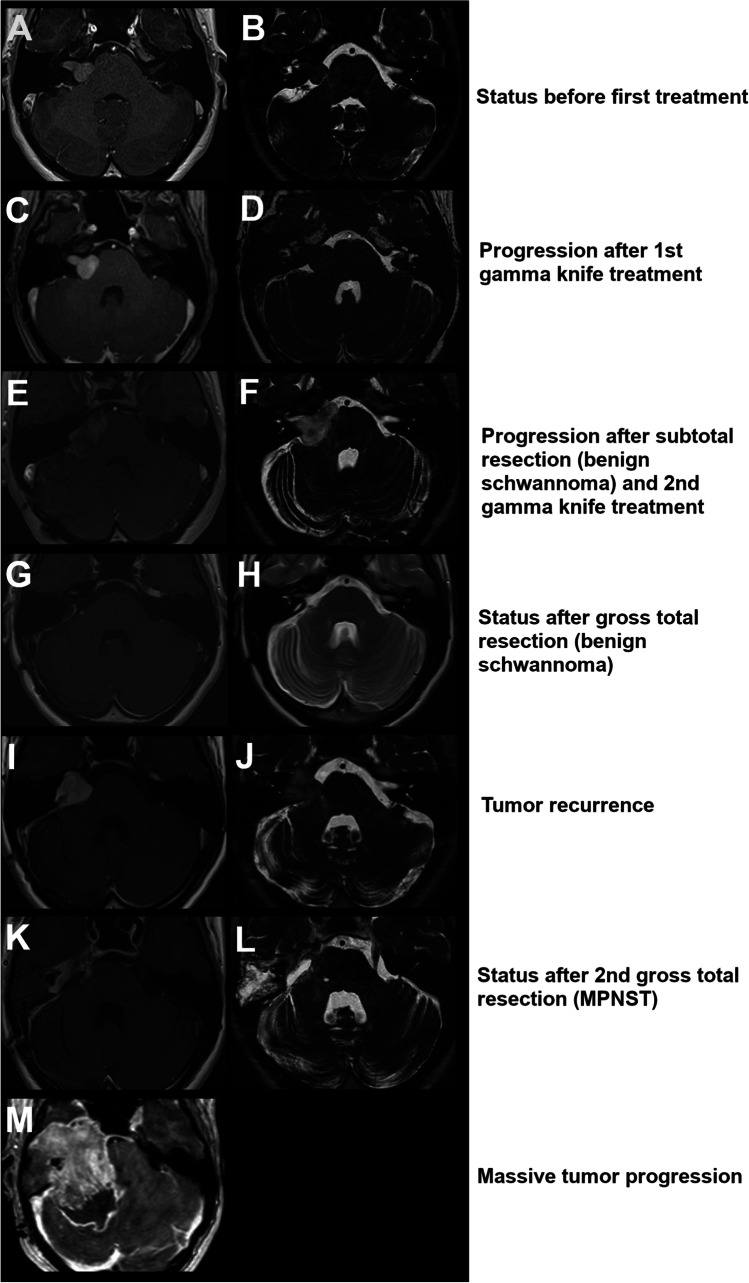
MR-images throughout the disease course showing the extent of the tumor prior to the first treatment with gamma knife (**A** and **B**) and further tumor growth 2 years later (**C** and **D**). Subsequently, the tumor was partially resected and after further growth a second round of gamma knife was applied. Afterwards, the tumor showed further progression (**E** and **F**) and gross total resection was done (**G** and **H**). Two years later, another tumor recurrence occurred (**I** and **J**) prompting another gross total resection now revealing the histology of a MPNST (**K** and **L**). Besides all efforts, the tumor showed a massive progression (**M**) and the patient deceased shortly afterwards

A 29-year-old woman was diagnosed with a right sided vestibular schwannoma due to progressive right-sided tinnitus and dizziness. The initial size was T3a according to the Hannover classification (Fig. 1A and B). Tumor growth was observed during the following 4 years (Fig. 1C and D) and the first treatment was performed with gamma knife radiosurgery at the age of 33. The target margin dose was 12 Gy to a 50% Isodose line covering a target volume of 1,3 cc. In the following months, the patient reported only an increase in tinnitus and in the further course also suffered dizziness and neuralgia in the territory of I and II right trigeminal branches that partially improved with Pregabalin. Further tumor progression was seen and the patient underwent partial resection at the age of 35. She required transient ventricular drainage on 08/21/13 due to post-operative bleeding that caused moderate obstruction of the IV ventricle with hydrocephalus. No shunt placement was necessary. The patient was referred to treatment with Gamma Knife radiosurgery for the residual schwannoma.

At the time or retreatment, the patient showed hypoesthesia in the territory of the right trigeminal III branch, right masseter paresis and pain in the glossopharyngeal territory on right-sided corneal stimulation. The remaining neurological examination, including confrontational campimetry and fundus examination, was normal. The maximum dose achieved of the second radiosurgery treatment was 20.8 Gy with a coverage dose of 12.5 Gy in the 60% isodose. Critical structures were considered. The right facial nerve received a dose lower than 9 Gy, the right trigeminal nerve a coverage dose and the brain stem a dose lower than 10 Gy (receiving a dose > 8 Gy in a volume of 0.09 cc and a dose > 5 Gy in a volume of 0.05 cc). The volume of tissue included within the coverage isodose was 0.823 cc.

**Fig. 2:**
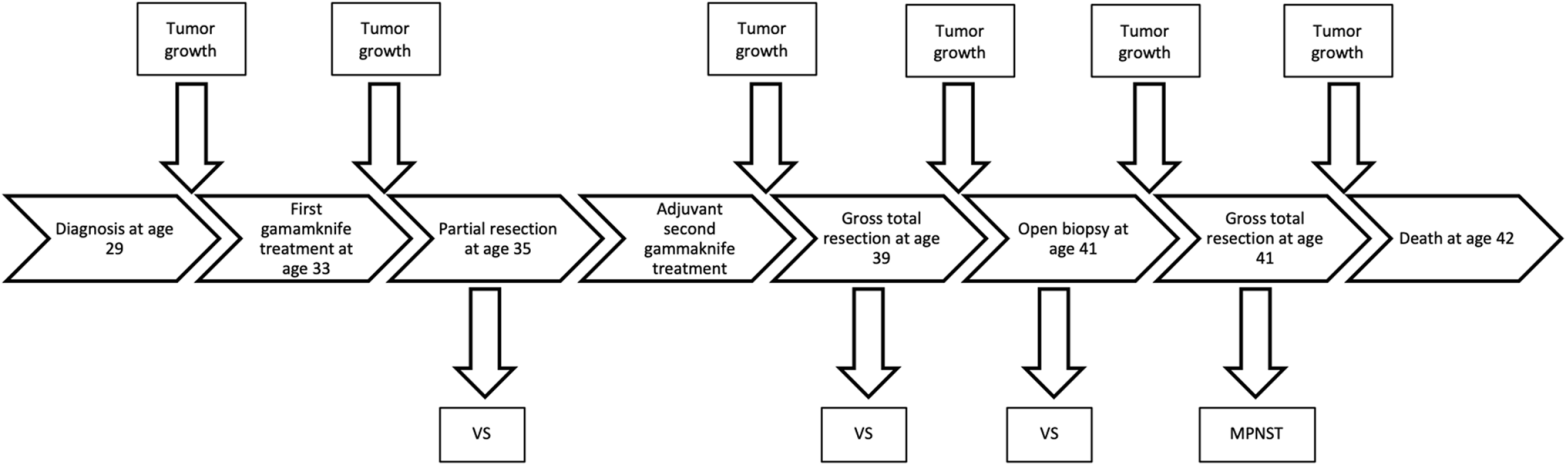
The histopathological images show the recurrent schwannoma after the gross total resection in our center as a moderately cellular spindle cell tumor without signs of atypia (**A**) and retained nuclear expression of H3K27me3 (**B**) (magnification 100 × . A biopsy of the next recurrence 2 years later revealed again a schwannoma. A spindle cell tumor of moderate cellularity consisting of plump neoplastic cells again with retained nuclear expression of H3K27me3 was seen (**C** and **D**) (magnification 100 ×). After the second gross total resection, we observed a highly cellular tumor of moderately atypical cells with signs of intraluminal vascular herniation (E, magnification 200 ×) and several mitotic figures (F, magnification 400 ×). Now, loss of nuclear expression of H3K27me3 in the tumor cells was detected, with positive endothelial cells, which served as an internal control (G, magnification 400 ×)

**Fig. 3:**
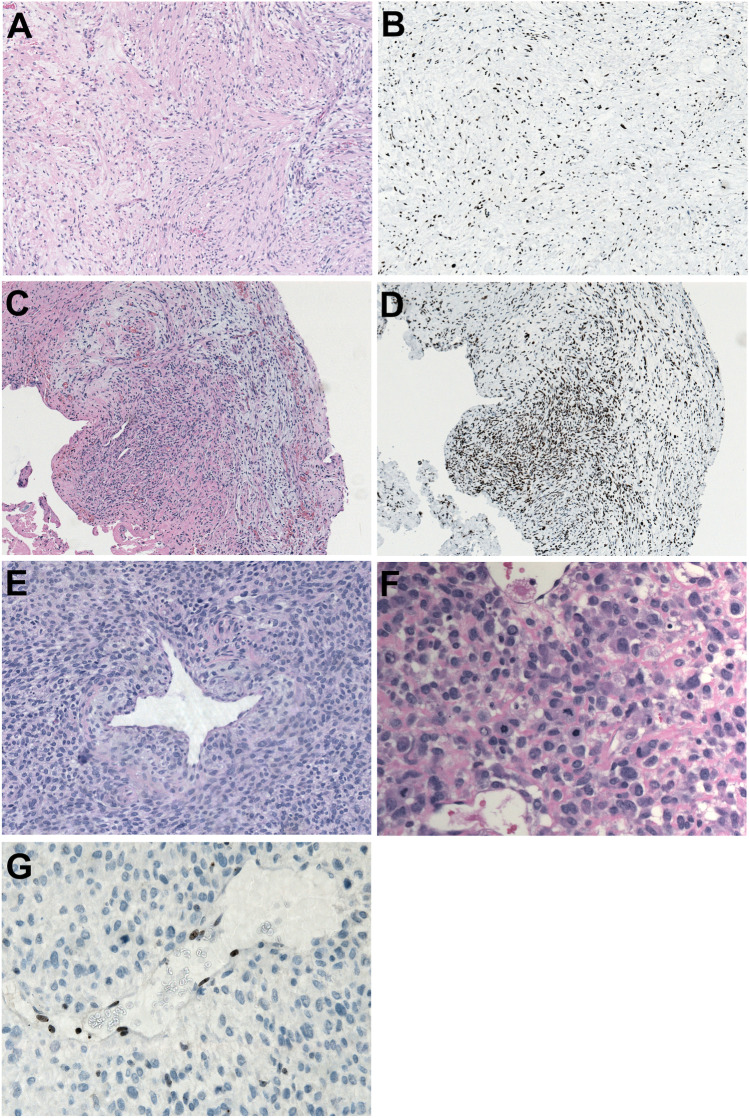
Timeline summarizing the course of disease and treatment

At the age of 39, the patient presented to our department with complete right-sided hearing loss, trigeminal neuralgia, periorbital spasm as well as balance disorders and mild dysphagia. The tumor had progressed to a T4a vestibular schwannoma (Fig. 1E and F). Gross total microsurgical resection was done with functional perseveration of the facial nerve with a postoperative facial palsy House & Brackmann grade III (Fig. 1G and H). The histopathology revealed a common schwannoma WHO grade I (Fig. 2A and B). Two years later at the age of 41, the patient presented to our department with another tumor recurrence which showed atypical imaging features (Fig. 1I and J). A change in histopathology towards malignant transformation was suspected and open microsurgical inspection and sampling offered. During surgery, severe adhesions of the tumor to the facial nerve were observed and biopsy showed a schwannoma WHO grade I (Fig. 2C and D). Due to further rapid growth within 8 months, radical surgical resection of the tumor including the infiltrated facial nerve with facial reconstruction was planned. During surgery infiltration of the brain stem was observed and another gross total resection was achieved (Fig. 1K and L). Now the histopathological evaluation showed the diagnosis of a malignant peripheral nerve sheath tumor (MPNST, Fig. 2E–G). Two days after surgery, a localized hematoma in the resection cavity was evacuated. The patient recovered quickly and was discharged one week after the revision surgery with a Karnofsky Performance Score of 80%. The case was presented and discussed in our neurooncological tumor board and the sarcoma board. Adjuvant radiation was recommended. Four months later, a massive tumor progression with rapid clinical decline occurred (Fig. 1M) and the patient died shortly afterwards at the age of 42. The disease course is summarized in Fig. 3

## Pathology findings

In the initial resection specimen, we observed the typical histology of a conventional schwannoma with a biphasic architecture consisting of moderately cellular, compact Antoni A regions alternating with loosely arranged Antoni B areas in roughly equal proportions. There were no discernible atypical features with tumor cells showing normochromatic elongated tapered nuclei with retained expression of H3(K27me3) (Fig. 2A and B).

Microscopic assessment of small tissue fragments from the recurrent tumour 2 years later revealed a similar blunt schwannoma histopathology with only minimal proliferative activity and retained nuclear positivity for H3(K27me3) (Fig. 2C and D).

In the latest radical resection specimen 8 months afterwards, we observed a highly cellular spindle cell tumour with a hemangiopericytoma-like vascular pattern and a vaguely fascicular architecture. In certain areas, intraluminal vascular herniation of the tumour cells could be identified. The neoplastic cells manifested moderate atypia und increased proliferation with abundant mitotic figures corresponding to a high-grade malignant peripheral nerve sheath tumor (Fig. 2E and F). Sparse residual tissue of the original benign schwannoma was focally discernible. Immunohistochemistry revealed a complete loss of immunoreactivity for H3(K27me3) in the neoplastic nuclei (Fig. 2G). An extensive negativity for S-100 and a nuclear accumulation of p53 could also be identified. An antibody stain against neurofilament revealed numerous entrapped nerve fibers consistent with an infiltrative growth pattern.

## Discussion

Schwannomas are typically encapsulated tumor consisting of well-differentiated Schwann cells with Merlin or SMARCB1 inactivation [9]. Cellular schwannomas are well-known variants and their hypercellularity and loss of rhythmic growth pattern must not be confused with true malignancy [10]. The transformation of a vestibular schwannoma into a malignant peripheral nerve sheath tumor after radiosurgery has been described in several cases before, as listed in Table 1. On the contrary, two cases of spontaneous malignant transformation without irradiation from benign schwannoma to malignant peripheral verve sheath tumor have also been reported [11–13]. Therefore, role of radiation therapy in malignant progression has been argued [14]. Overall, the mechanism of malignant transformation remains unclear, but it is important to report every case to allow for a better understanding in the future.

**Table 1:** List of reported vestibular schwannomas with malignant transformation in the literature with corresponding clinical data

Author,year	Age,Sex	NF2	1^st^ treatment	RTx	Marginal dose (Gy)	Latency (y)	2^nd^ pathology	Survival (m)
Comey, 1998	44, M	N	RTx	GK	14.4	5.5	MTT	12
Noren, 1998	18, F	Y	RTx	GK	20	5	MTT	n/a
Pollock, 1998	n/a	n/a	RTx	GK	16	7	MTT	n/a
Thomsen, 2000	19, F	Y	RTx	GK	12	6	Sarcoma	24
Baser 2000	n/a	Y	RTx	n/a	n/a	n/a	MPNST	n/a
	n/a	Y	RTx	n/a	n/a	n/a	MPNST	n/a
	n/a	Y	RTx	n/a	n/a	n/a	MPNST	n/a
Hanabusa, 2001	51, F	N	S	GK	15 + 14	0.5	Sarcoma	4
Bari, 2002	28, F	Y	RTx	GK	15	4	MPNST	3
Shin, 2002	26, F	N	S	GK	17	6	MPNST	10
Ho, 2002	14, F	Y	RTx	FRT	18	0.6	n/a	0.5
McEvoy, 2003	22, M	Y	RTx	GK	15	2	n/a	3
Wilkinson, 2004	53, M	N	S	n/a	n/a	7	MPNST	n/a
Muracciole, 2004	61, F	N	RTx	GK	10 + 12	6	MTT	n/a
Kubo, 2005	51, M	N	S	GK	14	0.7	MPNST	12
Hasegawa, 2005	56, F	N	RTx	GK	12.7	5.7	MPNST	12
Maire, 2006 Markou, 2012	45, F	N	S	FRT	n/a	18	MPNST	n/a
Van Rompaey, 2009	53, F	N	S	SRS	12	8	MPNST	n/a
Yang, 2010	74, M	N	S	SRS	12.5	6	Sarcoma	1
Dematriades, 2010	27, M	N	S	GK	15	10	MPNST	6
Akamatsu, 2010	67, F	N	S	GK	12	7.5	MPNST	n/a
Tanbouzi, 2011	15, M	Y	RTx	GK	13.5	5	MPNST	3
Schmitt, 2011 Milligan, 2012	51, M	N	RTx	GK	12	7.5	Sarcoma	7
Puataweepong, 2012	34, F	N	S	FRT	n/a	6	MPNST	n/a
Yanamadala, 2013	46, F	N	S	GK	14	6	MPNST	12
Kuzmik, 2013	73, F	N	RTx	SRS	13	0.5	MPNST	2
Seferis, 2014	46, F	N	S	GK	12	5.5	MPNST	n/a
Se, 2016	49, F	N	RTx	GK	12.5	6	Sarcoma	6 (alive)
Simmermacher, 2017	39, F	N	RTx	FRT	n/a	13	MPNST	n/a
Frischer, 2018	n/a	N	RTx	GK	13	8	MPNST	9
Haq, 2019	54, M	N	S	GK	10	9	MPNST	9 (alive)
Peker, 2019	40, F	N	S	CK	n/a	n/a	MPNST	n/a
Tish, 2019	65, F	N	RTx	GK	12	12	MTT	10
Boucher, 2020	66, F	N	RTx	GK	12.5	17	Sarcoma	7
Sherry, 2020	28, F	N	S	SRS	n/a	15	Sarcoma	n/a
	68, M	Y	RTx	GK	n/a	3	MPNST	12
Present case	29, F	N	RTx	GK	n/a	n/a	MPNST	4

*M* male; *F* female; *NF2* neurofibromatosis type 2; *n/a* not available; *S* surgery; *RTx* radiation therapy; *SRS* stereotactic radiotherapy; *FRT* fractionated radiotherapy; *GK* gamma-knife radiosurgery; *CK* cyber-knife radiosurgery; *MTT* malignant triton tumor; *MPNST* malignant peripheral nerve sheath tumor

In a series of 45 VS treated with fractionated radiotherapy, malignant transformation was seen in a case after long follow up which prompted the authors to suggest cautious use of radiation in young patients [15]. The case currently presented was 29 years old at the time of diagnosis and 33 at the first radiosurgery treatment. Looking at all reported cases so far [6–8, 15–44] (Table 1) patients of all age groups seem to be able to develop an MPNST after radiation, but it is remarkable that 10 out of 30 cases with age information were younger than 30 (33.3%). In comparison, the series of our department with 1381 vestibular schwannomas included only 202 patients of that age category (14.6%). We recently showed in a retrospective analysis that younger patients, suffering from sporadic VS, had larger tumor volumes and higher volumetric tumor growth rates prior to surgery [45] suggesting that sporadic VS developing at a younger age may behave differently. Furthermore, a more cautious indication for radiosurgery for vestibular schwannomas in NF2 patients has already been suggested [8].

Overall, longer follow-ups of large case numbers treated with radiosurgery, that can be expected in the near future, will shed more light on this rare but severe complication and hopefully allow for a risk stratification before treatment.

What is special about this current case is the detailed information about the H3K27 trimethylation status over the disease course. The loss of the trimethylation of lysin 27 of histone 3 is an increasingly established marker for the differentiation of MPNSTs from other pathologies [46]. The immunohistochemical detection of the loss of H3K27me3 has also been shown to be a highly sensitive marker for radiation induced MPNSTs [47]. Furthermore, MPNST with H3K27me3 loss clearly show a unique methylation profile that is clearly distinct from conventional and cellular schwannoma [48]. In this current case, we documented the course of H3K27me3 status with malignant tumor transformation. While the trimethylation of H3K27me3 was retained in the sample of the first gross total resection (Fig. 2B) and the subsequent biopsy that was done after further quick tumor progression (Fig. 2D), a clear H3K27me3 loss was detected in the last tumor sample that revealed the MPNST (Fig. 2G). Thus, the diagnostic role of H3K27me3 is likely transferrable to vestibular schwannomas, but may be influenced by tumor location, as H3K27me3 seems to be retained in spinal MPNST [48]. Immortalized Schwann cells do not show any reduction in H3K27me3 expression [49]. However, there is currently no information about the distribution of histone methylations in VS [50]. Future studies of H3K27me in larger cohorts of vestibular schwannomas are warranted.

MPNST arise usually in the setting of plexiform neurofibromas in NF1. In this case, we did not have any evidence of hereditary NF1 disorder or histological criteria associated with atypical neurofibromatous neoplasms of uncertain biologic potential (ANNUBP) [51].

## Conclusion

Malignant transformation of vestibular schwannoma to malignant peripheral nerve sheath tumor after radiation therapy is rare but fatal. Younger age seems to be a risk factor. The loss of the H3K27 trimethylation can be considered as a marker for malignant transformation of vestibular schwannoma.

## Data Availability

Not applicable.
